# Late-Onset Dilated Cardiomyopathy in Auto Immune-Mediated Complete Congenital Heart Block: A Case Report

**DOI:** 10.7759/cureus.54222

**Published:** 2024-02-14

**Authors:** Amulya Dharmagadda, Sampada Tambolkar, Sanjay Chavan, Srinija Garlapati

**Affiliations:** 1 Pediatrics, Dr. D. Y. Patil Medical College, Hospital and Research Centre, Dr. D. Y. Patil Vidyapeeth (Deemed to be University), Pune, IND

**Keywords:** pediatrics & neonatology, dilated cardiomyopathy, sjogren's, atrioventricular block, congenital heart block

## Abstract

Complete congenital heart block (CHB), a rare and fatal bradyarrhythmia observed in children, carries significant mortality and morbidity. When congenital heart block occurs in isolation with a structurally normal heart, it prompts suspicion of an autoimmune etiology, wherein maternal antibodies are transmitted transplacentally, impacting the fetal conducting system. The manifestation of congenital complete atrioventricular block (CCAVB) can lead to complications such as dilated cardiomyopathies, arrhythmias, and fibroelastosis in certain cases. Notably, dilated cardiomyopathy is a significant prognostic factor in children diagnosed with congenital heart block. Pathological investigations have revealed the presence of antibodies, complements, and indicators of inflammation or fibrosis across the myocardium, emphasizing the shared molecular mechanisms between CCAVB and the development of dilated cardiomyopathy (DCM). This article presents the case of a one-year-old female child who presented with signs of dilated cardiomyopathy, later identified through retrospective evaluation as having autoimmune congenital heart block. The mother of the child was diagnosed with Sjogren's syndrome, characterized by positive anti-RO titers. Remarkably, the child remained asymptomatic for a year without the need for pacing intervention. The child's condition was successfully stabilized with appropriate treatment, and plans for pacemaker insertion will be considered once specific criteria are met. The onset of cardiomyopathy in a known case of CCAVB should serve as a crucial alert for prognostic considerations and the potential necessity for early-pacing intervention.

## Introduction

Cardiac conduction anomalies are rarely reported in neonates and children, with an estimated prevalence of 1 in 15,000 to 20,000 live births [1,2]. The global incidence of dilated cardiomyopathy in the context of congenital heart block (CHB) ranges from 15% to 30% [3]. This condition may manifest in the heart that is structurally normal or in conjunction with congenital heart disorders.

The classification of CHB as congenital is attributed to the diagnosis either in utero, at birth, or within the initial month of life. Conversely, childhood block refers to cases identified beyond the first month up to 18 years of age [4]. The severity of the condition depends upon the degree of the block, categorized as a first-degree block, second-degree block (Mobitz type I or Wenckebach; Mobitz type II), and third-degree (complete) heart block [5].

Autoimmune congenital heart block refers to an immune-mediated cardiac disorder wherein maternal antibodies, specifically anti-Ro/La, are transmitted across the placenta, leading to the obstruction of atrioventricular (AV) node conduction despite the heart being structurally normal [4]. Newborns affected by congenital heart block primarily present with bradycardia while maintaining stable hemodynamics. In cases where Sjogren's disease is associated with congenital heart block, the predominant manifestation is often third-degree isolated complete heart block [6]. This severe form of heart block can result in fetal or infant mortality, therefore necessitating pacing intervention either during the newborn or at a subsequent stage of development.

Complete fetal AV block develops during 16 to 24 weeks of gestation [7]. Fetal echocardiography is the gold standard investigation for diagnosing congenital AV block and should be done as early as fetal bradycardia is detected [8]. Identifying an atrioventricular (AV) block in its early stages, specifically with a first-degree block, exhibits a more favorable prognosis and is potentially reversible with prompt intervention. In children diagnosed with CCAVB, the progression to dilated cardiomyopathy (DCM) is often associated with left ventricular (LV) dysfunction observed before pacemaker insertion [9]. This LV dysfunction is likely a consequence of an autoimmune mechanism triggered by the injury to the fetal cardiac conducting system by SSA/Ro and SSB/La antibodies [10, 11]. While the manifestation of AV block in this context is well-documented, the associated development of DCM has been studied to a lesser extent.

Unlike acquired AV conduction block, CHB identified prenatally in a structurally normal heart is associated with a distinct prognosis, carrying elevated risk for later developing dilated cardiomyopathy.

## Case presentation

**Figure 1:**
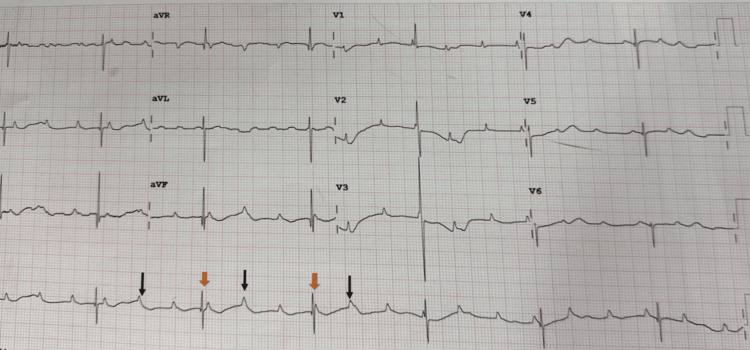
ECG showing complete congenital heart block Black arrows indicate P waves, and orange arrows indicate QRS complexes, which are independent of P waves.

A one-year-old female child presented with symptoms of fever, abdominal distension, and increased respiratory effort. Upon admission, bradycardia was observed, with a heart rate of 43 beats per minute (BPM). However, palpable pulses were noted, and no hypotension was evident (blood pressure: 90/60 mmHg). Peripheral oxygen saturation was 96% on room air, accompanied by noticeable intercostal and subcostal retractions and nasal flaring. An electrocardiogram (ECG) revealed a third-degree atrioventricular block, with an atrial rate of 125 BPM and a ventricular rate of 42 BPM (Figure 1). A 2D echocardiogram (2D ECHO) was conducted, indicating a structurally normal heart with moderate mitral regurgitation (MR), mild tricuspid regurgitation (TR), dilation of all four chambers, severe biventricular dysfunction, and a left ventricular ejection fraction ranging from 25% to 30%.

Other causes of dilated cardiomyopathy were ruled out in this child. Prenatal history revealed that the child had a confirmed case of complete congenital heart block. On detailed antenatal history taking, it was found that antenatal ultrasound performed at 8 months of gestation showed complete heart block and ventricular dysfunction in the fetus, despite having a structurally normal heart, along with a bradycardia of 56 beats per minute (BPM) then, a mother was diagnosed with Sjogren's with Anti RO/SSA (soluble substance A) IgG titers of 191.08 for which no treatment was taken. History also revealed that the mother had complete congenital blindness and hypothyroidism, besides a newly diagnosed autoimmune condition. The mother had received hydroxychloroquine four days before delivery and a full course of steroids (injection betamethasone) to promote lung maturation. The baby was delivered to a 33-year-old primigravida via cesarean section at 35.5 weeks of gestation, weighing 2100 grams. Although no resuscitation was required, the newborn was admitted to the neonatal intensive care unit (NICU) due to complete congenital heart block and prematurity. The infant was started on dobutamine, which was gradually tapered and discontinued over three days. Respiratory support was not necessary, and a 2D echocardiogram revealed a patent ductus arteriosus (PDA) measuring 2.8mm, in addition to bradycardia with no biventricular dysfunction. Feeding was gradually introduced over the subsequent 4 to 5 days, and the infant was discharged with supplement feeding on the 9th day of life. The child, previously diagnosed with autoimmune congenital heart block, now presented with one of its complications-dilated cardiomyopathy. The child had been asymptomatic until then and was developmentally appropriate for their age, but signs of failure to thrive were observed. Treatment involved the initiation of anti-diuretics (furosemide infusion, spironolactone), ionotropic support, and appropriate antibiotics and antivirals for an associated infection. The child's condition stabilized over a week and was subsequently discharged. Regular follow-up appointments with a pediatric cardiologist have been established, with plans for pacemaker insertion in the future once the child achieves a weight within the range of 15 to 20 kilograms.

## Discussion

Congenital heart block contributes to substantial mortality (20-30%) and morbidity (60-70%). During pregnancy, maternal autoantibodies cross the placenta and directly bind to calcium channels (L-type) on fetal myocardiocytes. Initially, this binding caused reversal inhibition of AV node conduction. However, prolonged exposure to these antibodies triggers the apoptotic pathway, leading to cell death and initiating inflammation locally. If the condition remains unchecked at this stage, the damage progresses, ultimately leading to calcification and fibrosis of the cardiac conduction system. This phenomenon has been named the "calcium channel hypothesis," which is currently regarded as the most accepted theory elucidating the pathogenesis of congenital heart block [1].

Usually, AV block manifests in fetuses of asymptomatic mothers who carry antibodies silently. Typically, the diagnosis is made retrospectively following the detection of fetal bradycardia. While fetal echocardiography is widely regarded as the gold standard for AV block diagnosis [12], it may be inadequate to identify early first-degree block, a milder form of the condition where intervention may not be necessary, or progression to complete block can be controlled through advanced treatment methods such as transplacental therapy involving fluorinated steroids. Recent non-invasive tools, namely fetal electrocardiography and fetal magnetocardiography, have aided greatly in overcoming these diagnostic challenges.

The majority of the instances with AV block can result in long-term sequelae, encompassing immune-mediated myocardial injury or transient myocarditis and cardiomyopathies. DCM in CHB prevails in almost 15% to 30% [13,14]. Without the implementation of pacemakers, this process is driven by a low heart rate accompanied by higher stroke volumes. Children having isolated CCAVB are susceptible to developing DCM, and risk factors may involve an early elevated cardiothoracic (CT) ratio, left ventricular dilation, and ventricular size showing minimal or no improvement with pacing. Despite positive maternal autoantibodies being commonly present in both CCAVB and CCAVB/DCM groups (79% and 87%, respectively), it is a debatable explanation for DCM development. In certain patients, dilated cardiomyopathy may be attributed as an associated condition of isolated CCAVB [4]. These significant issues hold critical importance during the diagnosis, as DCM at any life stage carries a poor prognosis.

Ventricular escape rates below 55 bpm and the presence of fetal hydrops elevate the risk of fetal and infant mortality [15]. During the fetal period, therapeutic interventions include transplacental treatments with steroids. Dexamethasone, administered to mothers, is occasionally used either independently or in conjunction with plasmapheresis and intravenous immunoglobulin (IVIG) [16]. Although the use of IVIG is subject to debate, it seems to significantly reduce fetal mortality. Terbutaline administration maternally has also been recommended in several reports [17]. For postnatal management, recommended treatments involve intravenous isoproterenol, dopamine, epinephrine, and atropine [18]. Pacemaker implantation is indicated in cases of symptomatic, non-reversible AV node disease and non-symptomatic high-degree AV blocks with specific risk factors.

## Conclusions

While being rare, congenital AV block is a significant treatable factor contributing to morbidity and mortality in children. There is a considerable need for further research, particularly in the area of antenatal detection and management, as well as on a genetic level. Despite varied etiologies necessitating specific management approaches and resulting in distinct outcomes, patients can achieve favorable outcomes with a meticulous and well-thought-out strategy in the diagnostic evaluation and management plan. The primary contributors to early mortality in CHB leading to DCM often stem from the delayed initiation of pacing therapy or instances of hemodynamic instability. Consequently, close monitoring and ongoing follow-up are imperative. This article also emphasizes maternal history, which is crucial in identifying risk factors that can contribute to present illness and varying treatment patterns accordingly.
